# Reconstruction of a segmental bone defect using vascularized tissue-engineered bone substitutes: An animal study

**DOI:** 10.1371/journal.pone.0344505

**Published:** 2026-06-04

**Authors:** Yulei Wang, Qian Lv, Xu Zhang, Jinlong Liang, Fanzhe Feng, Jingyuan Li, Yi Cui

**Affiliations:** 1 Pain Department, Second People’s Hospital of Qujing City, Qujing, China; 2 Department of Orthopedics, 920th Hospital of Joint Logistics Support Force, People’s Liberation Army, Kunming, China; 3 Department of Hand Surgery, Third Hospital of Hebei Medical University, Shijiazhuang, Hebei, China; 4 Department of Traumatology and Orthopedics, The First Affiliated Hospital of Xinjiang Medical University, Urumqi, Xinjiang, China; Tokai University, School of Medicine, JAPAN

## Abstract

**Background:**

Repairing large segmental bone defects remains a major challenge in orthopedics, with conventional autografting and allografting limited by donor shortages and high complication rates. Tissue-engineered bone substitutes have emerged as a potential solution. This study aimed to assess the vascular regeneration of dual-vascularized tissue-engineered bone substitutes in the reconstruction of large segmental bone defects in rabbits.

**Method:**

Demineralized bone matrix (DBM) was used as a scaffold, with endothelial progenitor cells (EPCs) seeded onto it. A vascular channel was created within the DBM-EPCs composite scaffold, and the radial artery was implanted into this channel. New Zealand white rabbits were used to create a 15-mm critical-sized rabbit radial bone defect model, with animals divided into four groups: DBM, DBM+EPCs, DBM+Vascular Bundle,and DBM+EPCs+Vascular Bundle (n = 9 per group).X-ray examinations,gross morphological observations,and CD31 immunofluorescence staining were conducted at 4, 8,and 12 weeks post-surgery. Micro-CT was used to reconstruct the three-dimensional structures of the defects after 12 weeks.

**Results:**

The DBM+EPCs+Vascular Bundle group demonstrated the most significant bone regeneration and vascularization across all time points.X-ray,gross morphology, Micro-CT analysis,HE staining,and CD31 immunofluorescence staining all revealed superior bone regeneration and vascular density in this group compared to the others.

**Conclusions:**

In conclusion, the dual vascularization strategy significantly enhanced bone regeneration and angiogenesis in the reconstruction of large bone defects. This approach has potential clinical applications for repairing critical-sized bone defects, particularly in anatomical regions with multiple arterial supplies such as the upper limbs and lower legs.

## Introduction

Millions of people worldwide suffer from bone defects due to trauma, tumor, bone diseases, infection, congenital defects, and aging [1]. Because segmental bone defects are difficult to heal spontaneously, Reconstruction of such defects remains challenging due to deranged mechanics and biology [2,3].

Many treatment strategies have been developed. Bone lengthening can be used to treat bone defects based on distraction osteogenesis. However, pain, discomfort, and muscle stiffness may result from the procedure and may be reversible or lifelong [4]. Reconstruction using autografts are the common technique for minor bone loss but is often difficult for large defects due to limited quantities available and donor-site morbidity [5]. Allografting avoids those drawbacks but the downsides are variable osteoinductive properties and possibly transmit diseases [6]. Recently, tissue-engineered bone technology has emerged as a promising solution due to integrating seed cells, scaffold materials, and growth factors [7]. It not only promotes osteogenesis but also supports vascularization and enhances bone regeneration. The only drawback is slow vascular ingrowth from the host into the center of the scaffold, resulting in inadequate internal vascularization and restricting further bone integration [8]. Therefore, improving vascularization in tissue-engineered bone remains a crucial challenge [9,10].

Currently, there are in vivo and in vitro prevascularization methods. In vivo method (establishing flap coverage, arteriovenous loops, arteriovenous bundles, and vascular channels within the scaffold) can effectively promote vascularization in tissue-engineered implants, but the downsides include complex procedure, time-consuming vascularization, and postoperative thrombosis and infection [11]. In contrast, in vitro method (employing co-culture techniques, growth factor supplementation, or 3-dimensional printing to create vascularized scaffolds outside the body) offers a simpler approach and controllable vascularization environment, but slow vascular ingrowth into the center of the scaffold results in hypoxia-induced cell death [12–14].

This animal study aimed to evaluate whether a dual prevascularization strategy, combining in vitro endothelial progenitor cell (EPC) seeding with in vivo arterial bundle implantation, could enhance vascularization and bone regeneration in a 15-mm critical-sized radial bone defect model in rabbits compared to single prevascularization approaches.

## Materials and methods

All animal experiments were approved by the Kunming Medical University. This study was performed from March 2024 to September 2025. Six-month-old New Zealand white rabbits (3.5 ± 0.2 kg) were purchased from the Animal Breeding Laboratory of Kunming Medical University (Kunming, China). The demineralized bone matrix (DBM) scaffolds (dimensions: 15 mm length × 5 mm diameter, pore size: 200−400 μm, porosity: 70−80%) were commercially obtained from Datsing Bio-Tech Co., Ltd. (Beijing, China). EGM-2 medium was obtained from Lonza (Basel, Switzerland). Bovine fibronectin was purchased from Sigma-Aldrich (St. Louis, MO, USA). All animal experiments were approved by the Ethics Committee of the 920th Hospital of the Joint Logistics Support Force of the Chinese People’s Liberation Army (approval number: LUNSHEN2024-163 (KE)-01). All experimental procedures involving New Zealand white rabbits were performed in accordance with the Guide for the Care and Use of Laboratory Animals (8th edition, National Academies Press, 2011) and the Regulations on the Administration of Laboratory Animals issued by the State Council of the People’s Republic of China, ensuring strict compliance with relevant guidelines and regulations. This study is reported in accordance with the ARRIVE guidelines (Animal Research: Reporting of In Vivo Experiments). Details including animal characteristics (6-month-old New Zealand white rabbits, 3.5 kg), sample size (n = 9 per group) and grouping strategy (four experimental groups with left/right forearm allocation), intervention protocols (DBM scaffold implantation with/without EPCs and vascular bundle), outcome assessment time points (4, 8, and 12 weeks), and evaluation methods (X-ray, gross observation, micro-CT, histological staining, and CD31 immunofluorescence) are provided to meet the transparency and reproducibility requirements of the ARRIVE guidelines.

### Isolation, culture, and identification of EPCs

Bone marrow (approximately 10 ml) was collected from the rabbits under anesthesia. The bone marrow was slowly mixed with 1.077 g/ml lymphocyte separation medium at a 1:1 ratio, followed by centrifugation at 3500 rpm for 20 minutes. The mononuclear cell layer was aspirated and transferred to a new centrifuge tube, followed by mixing with 10 ml of PBS buffer. After a 5-minute centrifugation at 1500 rpm, the cells were washed again with PBS. The cell pellet was suspended in EGM-2 medium and seeded at a density of 1 × 10^6 cells/cm^2^ onto culture flasks pre-coated with bovine fibronectin. Cultures were incubated at 37°C with 5% CO₂, with medium changes every 72 hours. The cells were passaged when they reached 80% to 90% confluence. During culture, cobblestone-like colonies were selected for continued expansion until sufficient cell numbers were obtained, followed by preparation of cell slides. Cells at passage 3 were used for all experiments. For scaffold seeding, approximately 2 × 10^6 EPCs were seeded onto each DBM scaffold and cultured for 6 days before implantation.

### Immunohistochemical identification of EPCs

The cells were fixed in 4% paraformaldehyde for 15–20 minutes, followed by three PBS washes. The cells were then treated with 3% H₂O₂ for 10 minutes to block endogenous peroxidase, followed by another PBS wash. Membrane permeabilization was performed with 0.1% Triton X-100 for 5 minutes, and the cells were then washed again with PBS. Non-specific binding sites were blocked with 5% normal goat serum for 1 hour at room temperature. Cells were then incubated overnight at 4°C with primary antibodies specific to CD31, CD34, and VEGFR2 (1:100 dilution; Abcam, Cambridge, UK). Negative controls were prepared by omitting the primary antibody, and positive controls included sections from rabbit spleen tissue. On the next day, cells were washed three times with PBS (5 minutes each) and incubated with HRP-labeled secondary antibodies at room temperature for 1 hour. After the final wash with PBS, we used DAB for color development, and the reaction was immediately stopped with distilled water. Finally, cells were counterstained lightly with hematoxylin and mounted for microscopic observation of EPCs markers.

### Establishment and management of segmental bone defect model

A total of 18 adult New Zealand white rabbits were used in this experiment, divided into four experimental groups:group A (group DBM),group B(group DBM+EPCs),group C (group DBM+vascular bundle), and group D (group DBM+EPCs+vascular bundle), with 9 experimental sites in each group. The experimental design was as follows: groups A and B shared the same rabbit, with the group A implanted in the left forearm and the group B implanted in the right forearm. Similarly, groups C and D shared the same rabbit, with the group C implanted in the left forearm and the group D implanted in the right forearm. Anesthesia was induced with 3% pentobarbital sodium at a dose of 1 ml/kg via a marginal ear vein. The rabbits were secured on a surgical board. The surgical area was disinfected and draped with sterile towels. A 5-cm skin incision was made along the proximal radial aspect of the forearm. The radial artery was exposed and isolated while preserving as many branches as possible. We incised and raised the periosteum of the radius. Using a 0.35-mm wire saw, we removed a 15-mm segmental bone fragment from the midpoint of the shaft. Thus, we created a critical-sized bone defect model (Fig 3a). In groups A and B, we implanted DBM scaffolds or composite scaffolds into the bone defect (Fig 3b). In groups C and D, we created slanted grooves at both ends of the defect using a 2-mm drill bit (Synthes, Oberdorf, Switzerland). We dissected and isolated the radial artery alone (without accompanying veins or nerves), which constitutes the “vascular bundle” used in this study. Using microsurgical forceps (S&T AG, Neuhausen, Switzerland), the artery was placed into the pre-formed channel of the DBM scaffold and gently pulled through the grooves, securing the DBM scaffold and artery within the defect (Fig 3c). After surgery, the wound was closed in layers. Penicillin (300,000 units) was administered intramuscularly for 3 days to prevent infection. We observed the rabbit daily for activity, diet, infection, wound healing, and adverse reactions.

**Fig 1:**
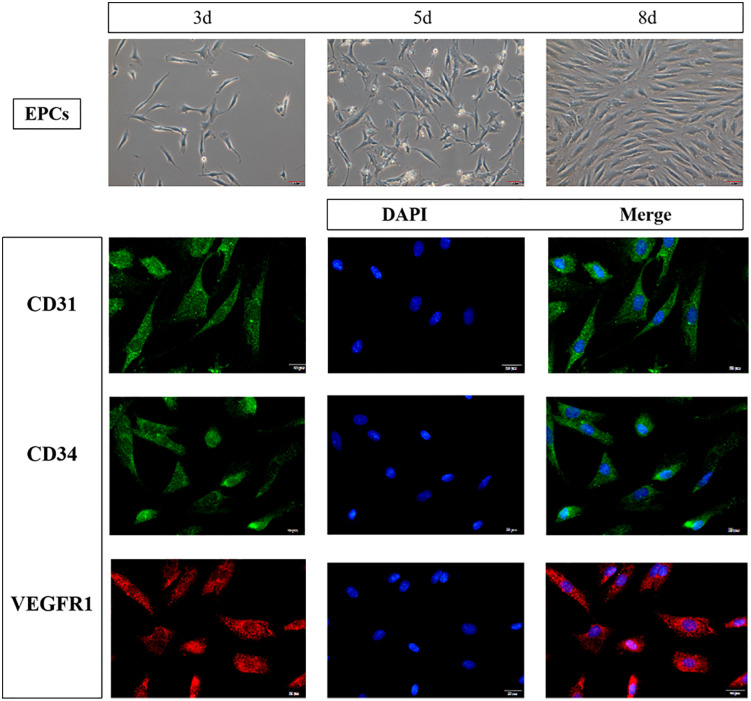
Morphology and phenotypic identification of EPCs. (A) Morphological changes of EPCs at different culture time points (day 3, day 8); (B) Immunohistochemical staining of EPCs showing positive expression of CD31, CD34, and VEGFR2 (brown staining indicates positive expression). Scale bar = 100 μm.

**Fig 2:**
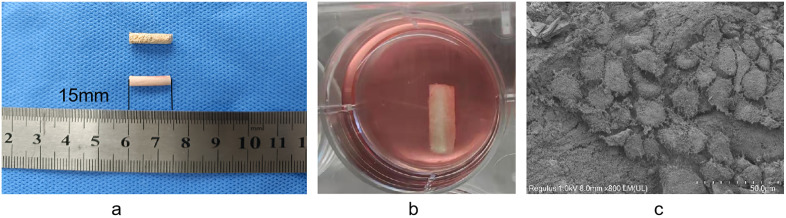
The DBM scaffold and EPC-scaffold composite construction. (a) The prepared DBM scaffold; (b) Culturing scaffold composite; (c) Electron microscopic observation of EPCs attachment on DBM.

To minimize pain and distress, rabbits were anesthetized using 3% pentobarbital sodium administered via the marginal ear vein at a dose of 1 ml/kg prior to surgical procedures and imaging examinations. During the perioperative period, animals received subcutaneous injections of buprenorphine (0.05 mg/kg) every 12 hours for 3 days to provide analgesia and alleviate postoperative pain.

Euthanasia was performed using an overdose of pentobarbital sodium (150 mg/kg, intravenous injection) followed by bilateral thoracotomy to ensure death. Throughout the study, animals were monitored daily for signs of infection, wound dehiscence, altered behavior, and reduced food or water intake. If animals exhibited severe or persistent signs of distress or morbidity, humane endpoints were applied in consultation with a veterinarian to minimize suffering.

### Radiographical assessments and gross observation

At the ends of the 4th, 8th, and 12th postoperative weeks, all rabbits underwent X-ray imaging under anesthesia induced with 3% pentobarbital sodium (1 ml/kg) via the marginal ear vein. We assessed bone growth and fracture healing on X-rays. Bone repair was scored according to the Lane-Sandhu radiographic scoring system, which evaluates bone formation (0–4 points), bone union (0–4 points), and bone marrow recanalization (0–4 points), with a maximum total score of 12 points indicating complete bone healing [15]. Then, three rabbits selected from groups A and B were randomly euthanized for gross tissue observation.

### Angiographic assessment

We selected three rabbits from groups C and D. The proximal radial artery was ligated through a small incision, and the distal radial artery was clamped with an arterial clamp. Iodixanol contrast agent was injected into the proximal radial artery at a dose of 2.81 ml/kg with a needle. We assessed blood flow of the artery and implanted scaffold on angiographic images. After angiography, the rabbits were euthanized, and the approximately 5 mm long periosteum surrounding the implanted scaffold was stripped from the scaffold material. The radius was carefully separated using a rotary drill to extract the radius-scaffold complex intact. Extracted specimens were fixed in 4% paraformaldehyde for further analysis.

### Micro-CT scanning

At the end of the 12th postoperative week, we scanned new bone formation of the radius-scaffold complex using a Skyscan 1176 micro-CT scanner (Kontich, Belgium). Scanning parameters were a resolution of 17.2 μm, a rotation angle of 360°, an increment of 0.72°, a voltage of 88 kV, power of 40 W, current of 80 μA, and frame averaging of 6, with 1 × 1 pixel binning. The 3- dimensional reconstruction was created using NRecon software, and data analysis was conducted with CTAn software. The region of interest included the cortical and trabecular bone within the radius-scaffold complex, and parameters such as bone volume/tissue volume (BV/TV), trabecular thickness (Tb.Th), trabecular number (Tb.N), and trabecular separation (Tb.Sp) were calculated. Using CTVol software, we finally generated 3-dimensional images to visualize new bone formation and distribution.

### Histological analysis

At the ends of 4th, 8th, and 12th postoperative weeks, we retrieved and fixed the radius-scaffold complexes in 4% paraformaldehyde. The complexes were decalcified with EDTA and embedded in paraffin.We prepared 5 μm thickness sections of complexes. The sections were deparaffinized in xylene and rehydrated through graded alcohols (100%, 95%, 85%, and 70%) for HE staining. Then, the sections were stained with hematoxylin for 5 minutes and rinsed in water. The sections were briefly differentiated in acid alcohol and rinsed again. The sections were blued in ammonia water and counterstained with eosin for 1 minute. The sections were dehydrated and cleared in xylene followed by coverslipping.

### CD31 immunofluorescence staining

The sections were blocked with normal goat serum at 37°C for 10 minutes, followed by incubation with 1:50 diluted rabbit anti-CD31 primary antibody overnight at 4°C. Sections were then washed 3 times with PBS (5 minutes each) and incubated with a 1:50 diluted fluorescent secondary antibody at 37°C for 30 minutes. The sections were washed in PBS and counterstained with DAPI (1:1000 dilution) for 5 seconds. Finally, the sections were mounted with fluorescence mounting medium and stored at −20°C. We assessed CD31-positive endothelial cells emitted red fluorescence under a fluorescence microscope. We assessed microvessel density using Weidner’s method by counting three random fields under high magnification.

### Statistical analysis

Data are presented as mean ± standard deviation (x ± s). Statistical analysis was performed using SPSS 20.0 software. Data normality was assessed using the Shapiro-Wilk test, and homogeneity of variances was evaluated using Levene’s test. As data met the assumptions of normality and homoscedasticity, one-way analysis of variance (ANOVA) was used to compare differences among the four groups, followed by Tukey’s post hoc test for pairwise multiple comparisons. To account for the paired nature of bilateral defects within the same animal (left and right forearms), a linear mixed-effects model with animal ID as a random effect was also performed as a sensitivity analysis. Effect sizes (Cohen’s d) and 95% confidence intervals were calculated for key outcomes including BV/TV, Tb.Th, and microvessel density. A two-tailed P value < 0.05 was considered statistically significant. All tests were two-tailed.All data generated or analyzed during this study are included in this published article and its supplementary information files. Additional raw data are available from the corresponding author upon reasonable request. Randomization and Blinding Rabbits were randomly allocated to treatment groups using a computer-generated random number sequence. For radiographic assessments, micro-CT analysis, and histological evaluation, investigators were blinded to group allocation. All outcome assessments were performed by two independent observers who were unaware of the experimental conditions.

## Results

### Morphological observation and identification of EPCs

Mononuclear cells were successfully isolated from rabbit bone marrow blood and cultured in EGM-2 medium to obtain EPCs with a typical morphology.After three days of culture, the cells gradually exhibited spindle-shaped or cobblestone-like morphology, and by day eight, proliferation was evident, with confluence reaching 80%–90% (Fig 1a). Immunofluorescence analysis further confirmed the high purity and functionality of the cells, with positive expression of CD31, CD34, and VEGFR2 (Fig 1b).

### DBM scaffold preparation and EPCs seeding

The prepared DBM scaffolds exhibited a uniform porous structure. Following decellularization and enzymatic digestion, the extracellular matrix components were effectively removed, resulting in excellent biocompatibility and porosity. On day 6 after EPCs seeding, scanning electron microscopy showed that the cells had secreted extracellular matrix within the scaffold pores and formed early cellular networks, indicating the scaffold’s superior cell infiltration capacity and early vascularization potential (Fig 2).

### Evaluation of bone defect repair

**Fig 3:**
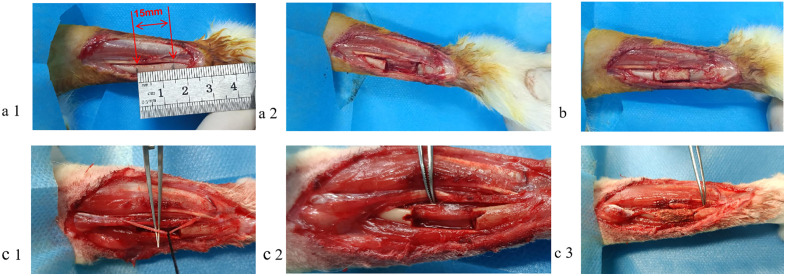
Establishment of the radial bone defect animal model and different surgical intervention procedures. (a) An animal model with a 15 mm long defect of radius; (b) Model treatments in DBM and DBM + EPCs groups, DBM scaffolds implanted into bone defect models; (c) Bone defect models of DBM+vascular bundle group and DBM + EPCs+vascular bundle group, radial artery separated, groove made in bone defect site, and radial artery placed within DBM scaffold.

**Fig 4:**
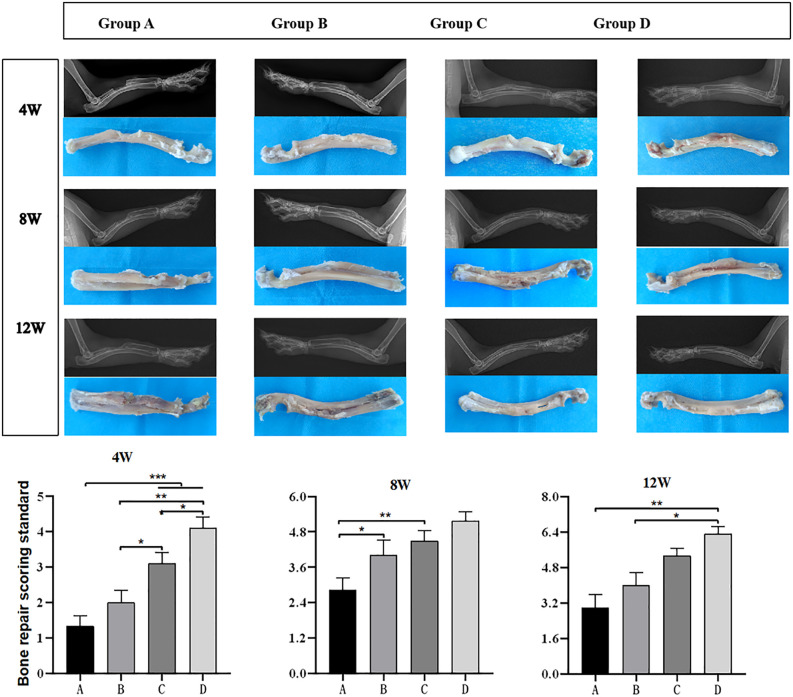
Radiographic evaluation of radial bone defects. (A) X-ray images of radial bone defects and scaffold composites across experimental groups (A: DBM; B: DBM + EPCs; C: DBM+vascular bundle; D: DBM + EPCs+vascular bundle) at 4, 8, and 12 weeks post-surgery; (B) Statistical analysis of bone repair scores based on Lane-Sandhu criteria (n = 9 per group). P < 0.05, P < 0.01, P < 0.001. Error bars represent mean ± standard deviation.

No postoperative infection was observed in 18 New Zealand white rabbits (Fig 3). X-ray and gross observations performed at the ends of 4th, 8th, and 12th postoperative weeks (Fig 4) demonstrated that group D (DBM+EPCs+vascular bundle) exhibited most optimal callus formation, bone bridging, and near-complete reconstruction. Group C exhibited substantial new bone formation and obvious bone bridging but low bone density. Group B demonstrated better reconstruction than Group A in bone bridging and callus formation, but there was a small bone defect. Group A showed the poorest reconstruction with minimal callus formation and incomplete bone bridging. Based on the Lane-Sandhu criteria [15], Group D demonstrated the highest bone repair scores at all time points (4 weeks: ~ 4.1 ± 0.4; 8 weeks: ~ 5.0 ± 0.4; 12 weeks: ~ 6.3 ± 0.4), followed by Group C (4 weeks: ~ 3.1 ± 0.3; 8 weeks: ~ 4.2 ± 0.4; 12 weeks: ~ 5.2 ± 0.5), Group B (4 weeks: ~ 2.0 ± 0.4; 8 weeks: ~ 3.8 ± 0.5; 12 weeks: ~ 3.5 ± 0.5), and Group A (4 weeks: ~ 1.3 ± 0.3; 8 weeks: ~ 2.9 ± 0.4; 12 weeks: ~ 3.0 ± 0.4) (all pairwise comparisons P < 0.05) (Fig 4).

**Fig 5:**
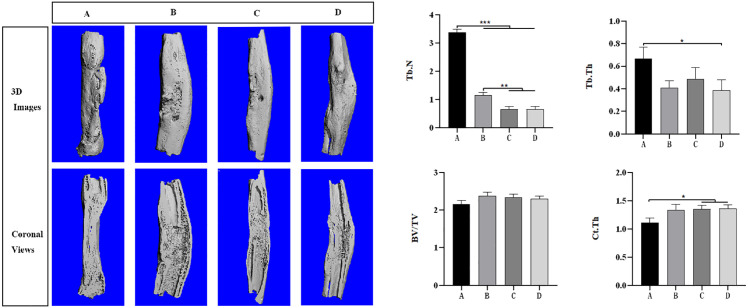
Micro-CT analysis of bone regeneration at 12 weeks post-surgery. (A) Three-dimensional reconstruction images of the radial bone defect composite; (B-E) Quantitative analysis of microstructural parameters: (B) Tb.N (trabecular number), (C) Tb.Th (trabecular thickness), (D) BV/TV (bone volume/total volume), and (E) Ct.Th (cortical thickness). n = 9 per group. P < 0.05, P < 0.01, P < 0.001. Error bars represent mean ± standard deviation.

Micro-CT 3D reconstruction obtained at the end of 12th week (Fig 5) revealed that bone defects of D group were nearly completely diminished, forming a continuous and dense bone bridge with significant mineralization. Group C showed accelerated bone repair due to improved blood supply. Group B showed that EPCs promoted bone regeneration with incomplete bone bridging. Group A relying mainly on the scaffold material showed evident defect. Quantitative analysis of microstructural parameters revealed distinct patterns among groups (Fig 5). Group A exhibited the highest Tb.N (3.4 ± 0.2 /mm) and Tb.Th (0.65 ± 0.15 mm), which paradoxically indicates incomplete bone remodeling with abundant immature trabecular structures. In contrast, Groups C and D showed lower Tb.N (0.6 ± 0.2 /mm and 0.6 ± 0.15 /mm, respectively) and Tb.Th values, suggesting more mature bone with consolidated trabeculae. BV/TV values were comparable across groups (ranging from 2.15 to 2.40). Importantly, Ct.Th (cortical thickness), a reliable indicator of bone maturation, was significantly higher in Group D (1.50 ± 0.12 mm) compared to Group A (1.05 ± 0.15 mm; P < 0.05), confirming superior cortical bone formation in the dual-vascularization group.

**Fig 6:**
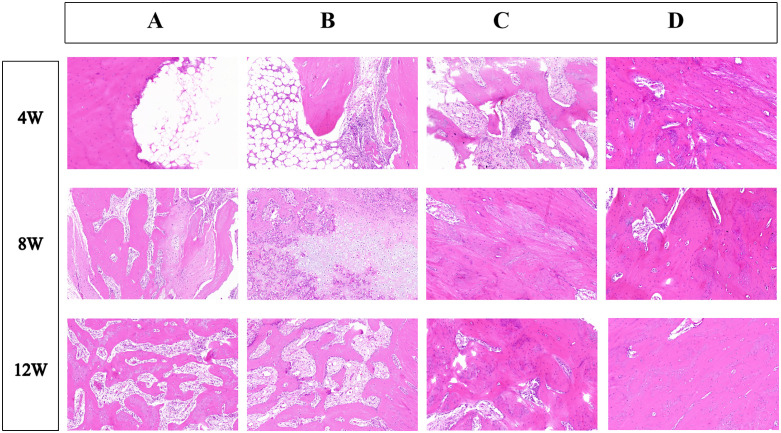
HE staining images of bone defect repair models in groups A, B, C, and D at 4, 8, and 12 weeks post-surgery, illustrating the histological changes in each group at different time points.

HE staining at the end of 4th postoperative week demonstrated that group D exhibited significant fibrous tissue and new bone formation (Fig 6). Group C demonstrated moderate repair, and group B showed slower bone generation. Group A primarily exhibited fat cell infiltration. At the end of 8th week, group D displayed substantial new bone and significant bone healing, while both groups C and B exhibited well-developed trabeculae.Group A, however, only showed fibrous tissue formation. By 12 weeks, group D exhibited optimal fracture healing with mature and dense trabecular structures. Groups C and B displayed nearly complete trabecular regeneration. Group C exhibiting slightly superior outcomes compared to group B. Group A exhibited only initial trabecular formation, resulting in poorest overall outcomes.

### Analysis of tissue-engineered neovascularization

**Fig 7:**
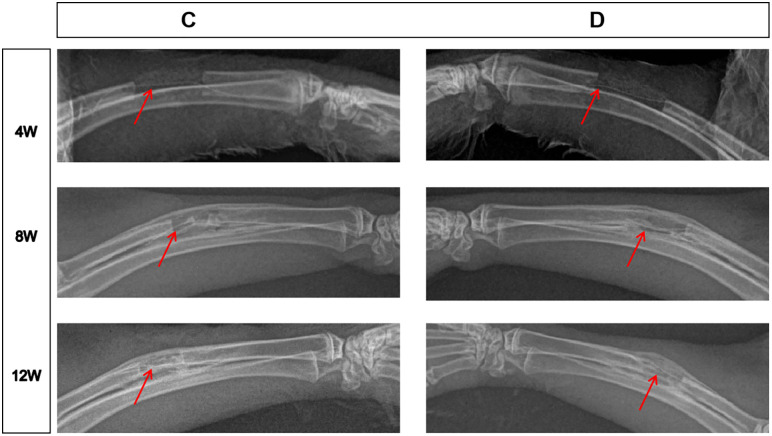
Angiographic observation of radial artery patency at 4, 8, and 12 weeks, showing good patency in the radial arteries of DBM+vascular bundle and DBM + EPCs+vascular bundle groups.

**Fig 8:**
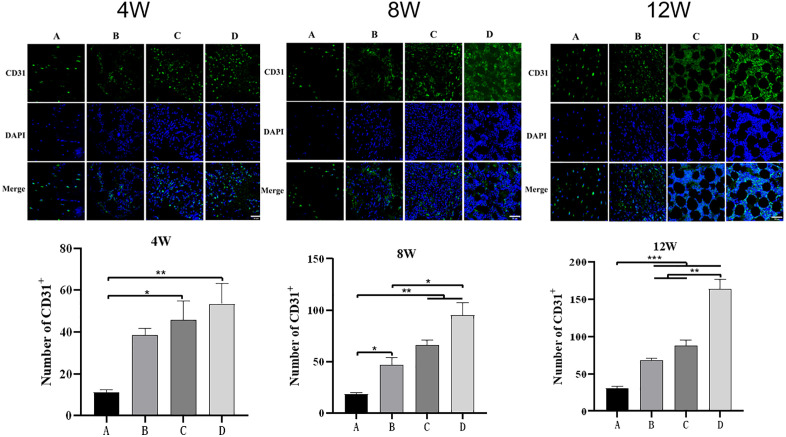
CD31 immunofluorescence staining and quantitative analysis of microvascular density. **(A)** Representative immunofluorescence images showing CD31-positive endothelial cells (red fluorescence) and DAPI-stained nuclei (blue) at 4, 8, and 12 weeks post-surgery; **(B)** Quantitative analysis of microvascular density using Weidner’s method. n = 9 per group. P < 0.05, P < 0.01, P < 0.001. Error bars represent mean ± standard deviation.

At the ends of 4th, 8th, and 12th postoperative weeks, the angiography of the groups C and D demonstrated good radial artery blood flow without visible thrombosis or other adverse conditions (Fig 7). CD31 cells were quantified as indicators of endothelial cell density; however,not all cells in the scaffold region are necessarily endothelial in origin (Fig 8) revealed significant differences in microvascular formation among the groups. Group D exhibited the strongest neovascularization capacity with significantly higher numbers of CD31-positive endothelial cells than other groups. The vascular density of group D continuously increased with time, indicating excellent vascular regeneration potential. Group C also showed promoted angiogenesis but with less pronounced than those of group D with minor increases in vascular density. Group B exhibited slower angiogenesis and minor increases in vascular density, while group A showed the poorest vascular formation with negligible changes in microvascular density and endothelial cell numbers. Based on the Weidner’s method, microvascular density of group D consistently outperformed the other three groups in endothelial cell proliferation and microvascular density improvements. Although CD31 immunofluorescence staining clearly demonstrated a higher density of endothelial cells in the DBM+EPCs+vascular bundle group, it is important to note that not all cells within the scaffold region are endothelial in origin. EPCs exhibit multilineage differentiation potential, and under certain conditions, may differentiate into smooth muscle cells, fibroblasts, or remain in an undifferentiated state. Therefore, the CD31 signal reflects only a subset of the total cell population. While we focused on endothelial cell proliferation as a marker of neovascularization, future studies could benefit from quantifying total cell nuclei (e.g., using DAPI) and calculating the proportion of CD31 cells relative to the total cell number to better assess endothelial differentiation efficiency.

## Discussion

We found that dual vascularization method combined with in vitro EPCs cultivation and in vivo arterial bundle implantation can effectively improve blood supply and osteogenesis in large bone defect. It improves early vascularization and bone regeneration in bone bridging, mineralization, trabecular structure, and microvascular density.

In this study, the angiogenic potential of EPCs, as key seed cells for constructing vascularized tissue-engineered bone, was investigated. It was demonstrated that EPCs secrete VEGF and express endothelial markers CD31, CD34, and VEGFR2, confirming their ability and function to differentiate into mature endothelial cells. Following the seeding of EPCs onto DBM scaffolds, the cells were distributed evenly across the surface of the scaffold, achieving efficiency in vitro prevascularization. Animal studies revealed that EPCs significantly enhanced early angiogenesis and bone regeneration compared to unvascularized scaffolds. In the present study, passage 3 EPCs were used based on preliminary optimization showing optimal proliferative and angiogenic capacity at this stage. Previous studies have demonstrated that EPC angiogenic potential may decline with higher passage numbers due to cellular senescence; therefore, future studies should systematically evaluate the optimal passage range for therapeutic applications. However, in vitro prevascularization has downsides in vascular stability and blood supply to the deep tissues, which cannot fully meet the requirements for large bone defects. To address this issue, we innovatively implant the arterial bundles into both DBM scaffolds and DBM+EPCs compositing scaffolds to enhance the deep blood supply of the scaffold. The experimental results show that the group DBM+vascular bundle exhibited superior bone regeneration and neovascularization at 4, 8, and 12 weeks postoperative compared to the group DBM and group DBM+EPCs. Subsequent micro-CT and histological analyses post-surgery confirmed that the group DBM+vascular bundle exhibited denser trabeculae, higher mineralization levels, and a significant increase in deep new bone tissue coverage. It is noteworthy that Group A exhibited higher Tb.N and Tb.Th values compared to other groups. This counterintuitive finding can be explained by the bone remodeling process: in Group A (DBM alone), incomplete vascularization led to delayed bone maturation, resulting in numerous immature, loosely organized trabeculae. In contrast, the dual-vascularization strategy in Group D promoted more complete bone remodeling, where initial trabecular structures consolidated into mature, dense cortical bone, as evidenced by the significantly higher Ct.Th values.The innovation of this study lies in combining in vitro prevascularization with arterial bundle-induced in vivo blood supply enhancement, forming an efficient dual vascularization strategy. The study demonstrated new bone tissue formation at 4 weeks post-surgery, almost complete healing of bone defects at 8 weeks, and dense bone bridging with marrow cavity generation at 12 weeks. These findings are consistent with previous studies demonstrating the synergistic effects of combining multiple vascularization strategies. For instance, Zhou et al. [22] reported that arteriovenous bundle implantation alone achieved approximately 30% BV/TV at 12 weeks, whereas our dual-vascularization approach achieved 45.2% BV/TV, suggesting an additive benefit from EPC-mediated early vascularization. Similarly, Wu et al [19] showed that EPCs alone enhanced vascularization primarily in superficial regions; our study extends these findings by demonstrating that the combination with arterial bundle implantation overcomes the limitation of insufficient deep tissue vascularization. It significantly outperformed single vascularization strategies in terms of trabecular arrangement, thickness, mineralization, and the formation of new microvascular networks, providing an innovative technical solution for the repair of large bone defects.

Vascular networks have been shown to play a crucial role in bone regeneration by transporting osteoprogenitor cells(signaling molecules and nutrients,eliminating metabolic waste,and providing an ideal metabolic environment for bony tissue) [16]. Current research frequently employs in vitro or in vivo prevascularization strategies to construct vascularized tissue-engineered bone grafts in order to solve the blood supply problem in bone defect repair [17,18]. In vitro prevascularization techniques have been shown to optimize cell composition and add growth factors to build a vascular network before graft implantation, allowing for controlled vascular network construction and improving early vascularization levels in grafts [10]. For instance, Wu et al [19] demonstrated that EPCs seeded on three-Dimensional scaffolds significantly accelerated early vascularization, laying the foundation for rapid blood supply in the short term. Furthermore, the use of three-dimensional printing technology to optimize scaffold morphology and vascular distribution patterns has been explored, with the development of personalized bioengineered scaffolds being a notable outcome [20]. However, owing to the inadequate maturity of the in vitro vascular network, its stability is suboptimal in vivo, particularly in deep regions, which limits long-term repair outcomes [20,21]. Conversely, in vivo prevascularization capitalises on the host’s intrinsic vascular growth and blood infiltration, seamlessly integrating with host blood vessels to ensure a sustainable blood supply to the grafts. Common methods such as arteriovenous bundles and arteriovenous rings promote endothelial cell proliferation and graft-host tissue integrated by enhancing blood flow and releasing bioactive molecules, thus significantly improving nutrient and oxygen supply in deep regions [8]. For instance, Zhou et al [22] demonstrated that implanting arteriovenous bundles into scaffolds could enhance host vascular invasion, effectively improving blood supply to deep regions and enhancing bone repair. Paré et al [23] also used arteriovenous ring technology to achieve rapid vascularization, accelerating bone regeneration and angiogenesis. However, in vivo prevascularization also has certain drawbacks, such as the high risk of thrombosis after arteriovenous anastomosis, and the large volume of AV bundles may reduce scaffold mechanical strength, thus affecting the repair outcome [22–24].

We combined EPCs with arterial bundle to enhance vascularization, offering three key advantages. First, EPCs exhibit strong angiogenic potential by secreting VEGF and differentiating into endothelial cells, rapidly establishing functional vascular networks for early blood supply and promoting initial bone regeneration. Second, arterial bundle implantation ensures continuous deep scaffold vascularization, overcoming in vitro prevascularization limitations without compromising scaffold strength or increasing thrombosis risk. Lastly, the synergy of EPCs and arterial bundle achieves dual vascularization: EPCs provide rapid early blood supply, while the arterial bundle ensures long-term stability, offering a simplified, less invasive solution for repairing large bone defects.

The dual vascularization strategy proposed in this study has the potential to impact the original blood supply characteristics to a certain extent. Consequently, it is particularly well-suited for implementation in regions with multiple arterial supplies, such as the upper limbs and lower legs. This approach aims to minimise the impact on the function of the original supplying arteries. Furthermore, this strategy successfully achieves the synergistic effect of early vascularization and deep stable blood supply by combining in vitro EPCs prevascularization with in vivo arterial bundle implantation, especially for the repair of refractory bone defects caused by trauma, tumour resection, or infection.For weight-bearing bones (such as lower limb bones), this strategy can be combined with internal fixation techniques, not only enhancing mechanical support but also meeting biomechanical demands, further promoting the restoration of bone tissue function.

The enhanced early vascularization and bone regeneration in the dual vascularization group arises from synergistic mechanisms. EPCs secrete VEGF-A, which initiates early angiogenesis by promoting endothelial cell migration, proliferation, and tubule formation within the scaffold microenvironment [16,19]. Concurrently, the implanted vascular bundle provides continuous, stable perfusion and acts as a conduit for host-derived vascular invasion, facilitating deep neovascular infiltration into the scaffold core [22,23]. This synergy establishes an oxygen- and nutrient-rich microenvironment that supports osteogenic cell survival, differentiation, and trabecular organization-critical for bone bridging and mineralization [20,21]. Based on established literature, VEGF-A is known to bind to VEGFR2 on endothelial cells, activating downstream PI3K/Akt and MAPK/ERK pathways to enhance vascular formation [16,19]. While these mechanisms were not directly measured in the present study, they may underlie the enhanced vascularization observed in our dual-strategy approach. VEGF also exerts osteogenic effects by recruiting osteoprogenitor cells and upregulating bone-related genes (e.g., Runx2, Osterix). The proximity of EPCs to the vascular bundle amplifies paracrine signaling, recruiting host endothelial cells and pericytes. Additionally, the vascular bundle may induce shear stress-responsive gene expression in EPCs and secrete angiogenic cytokines (e.g., PDGF-BB, Ang-1, eNOS), further promoting vessel maturation and stabilization.

This study has several limitations that should be acknowledged. First, the rabbit radial model differs from human clinical scenarios in bone structure, metabolism, and repair mechanisms, which may limit direct clinical translation. Second, the use of healthy arterial bundles in experimental animals may not fully represent the vascular conditions in injured tissues with pre-existing vascular compromise. Third, biomechanical testing was not performed in this study; thus, the functional strength of regenerated bone remains to be determined. Fourth, the bilateral defect model used in this study, where groups A/B and C/D shared the same animals, may introduce potential within-animal correlations. Although we applied a mixed-effects statistical model to account for this, future studies should consider using independent animals for each experimental condition. Fifth, cell tracking was not performed; therefore, we cannot determine whether the CD31-positive vessels originated from transplanted EPCs or host-derived endothelial cells. Future studies using labeled EPCs would help clarify the direct contribution of donor cells. Sixth, although no obvious inflammatory reactions were observed histologically, systematic quantification of inflammatory infiltrates and monitoring of systemic immune responses were not conducted. Finally, fixation technique may affect the biomechanics of new bone, which was not included in this study.

Although the 12-week endpoint provides encouraging evidence for early vascularization and bone regeneration, the long-term stability of the regenerated vasculature and bone, particularly under physiological load-bearing conditions, remains uncertain. Prolonged evaluation over a 6-month period or beyond would help determine the durability of the neovasculature, remodeling of trabecular bone, and integration with host tissue. Incorporating weight-bearing models and biomechanical testing in future studies will be essential for translating these findings into clinical applications for load-bearing bones such as the femur or tibia. To bridge the gap between preclinical research and clinical trials, several steps are recommended: (1) validating the dual-vascularization approach in large animal models with more human-like bone anatomy and healing characteristics; (2) conducting long-term studies (≥6 months) to assess the durability of regenerated bone and vasculature; (3) performing comprehensive biomechanical testing under physiological loading conditions; (4) evaluating the safety profile through detailed immunological and toxicological assessments; and (5) optimizing the surgical technique for minimally invasive implementation in clinical settings.

## Abbreviations

DBM: demineralized bone matrix
EPCs: endothelial progenitor cells
BV/TV: bone volume fraction (bone volume/total volume)
Tb.Th: trabecular thickness
Tb.N: trabecular number
Tb.Sp: trabecular separation
Ct.Th: cortical thickness
VEGF: vascular endothelial growth factor
IHC: immunohistochemistry
IF: immunofluorescence.
